# Simple Preparation of Novel Metal-Containing Mesoporous Starches †

**DOI:** 10.3390/ma6051891

**Published:** 2013-05-10

**Authors:** Manuel Ojeda, Vitaliy Budarin, Peter S. Shuttleworth, James H. Clark, Antonio Pineda, Alina M. Balu, Antonio A. Romero, Rafael Luque

**Affiliations:** 1 Departamento de Quimica Orgánica, Universidad de Córdoba, Campus de Rabanales, Edif. Marie Curie, Ctra. Nnal IV-A, Km 396, Córdoba E14014, Spain; E-Mails: b82ojrom@uco.es (M.O.); q82pipia@uco.es (A.P.); qo1rorea@uco.es (A.A.R.); 2 Green Chemistry Centre of Excellence, The University of York, Heslington, York YO10 5DD, UK, E-Mails: vitaliy.budarin@york.ac.uk (V.B.); james.clark@york.ac.uk (J.H.C.);; 3 Departamento de Física de Polímeros, Elastómeros y Aplicaciones Energéticas, Instituto de Ciencia y Tecnología de Polímeros, CSIC, C/ Juan de la Cierva 3, Madrid 28006, Spain; E-Mail: peter@ictp.csic.es; 4 Department of Forest Products Technology, School of Chemical Technology, Aalto University, P.O. Box 16300, Aalto FI-00076, Finland; E-Mail: alina.balu@aalto.fi

**Keywords:** mesoporous polysaccharides, starch, Fe, Co, Cu

## Abstract

Metal-containing mesoporous starches have been synthesized using a simple and efficient microwave-assisted methodology followed by metal impregnation in the porous gel network. Final materials exhibited surface areas >60 m^2^ g^−1^, being essentially mesoporous with pore sizes in the 10–15 nm range with some developed inter-particular mesoporosity. These materials characterized by several techniques including XRD, SEM, TG/DTA and DRIFTs may find promising catalytic applications due to the presence of (hydr)oxides in their composition.

## 1. Introduction

**Figure 1:**
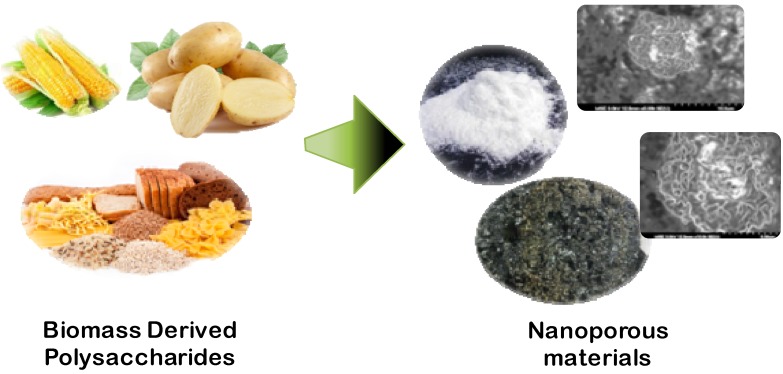
Overview of the research objective; transformation of non-porous native polysaccharide into useful porous carbonaceous materials. Adapted from [1].

Mesoporous materials prepared from renewable feedstocks have attracted a great deal of attention in recent years due to their promising applications in areas including water purification, heterogeneous catalysis, separation media and energy storage [1,2,3,4,5]. The development of novel porous media using benign, environmentally friendly and low environmental impact resource efficient technologies is a significant challenge. In this regard, biomass-derived materials hold significant potential to be converted into useful novel structures if the low mechanical/chemical resistance of native biomass is overcome (Figure 1) [1,2,3,4,5,6].

Recent research endeavours from the group have been directed to the development of novel families of polysaccharide-derived porous materials avoiding the use of templates in their preparation (e.g., from precursors including starch, pectin, alginic acid all the way to the so-called Starbon^®^ materials via carbonization [1,6,7,8]) and their application in fields including adsorption [9,10], antibacterial activity [11], separation [12,13] and catalysis [14,15]. These materials offer a greener alternative to conventional mesoporous templated formed materials, opening at the same time pathways to generate innovative porous materials with different structures [1,6]. The developed methodology involves a simple expansion of the native biopolymer via aqueous gel formation upon heating, retrogradation (cooling) followed by solvent exchanging and drying [1,6]. Subsequent thermal treatment of these solid mesoporous polysaccharides renders Starbon^®^ materials which possess varying textural and structural properties depending on the carbonization temperature and can be further functionalized with a range of functional groups (e.g., acid and basic sites, nanoparticles, *etc.*) [1,6,14,15]. Functionalization of porous polysaccharides to useful products is difficult due to the labile nature of polysaccharides to acid/base or redox conditions (e.g., starch) [16,17].

Template-free Porous polysaccharides can be metal-functionalized, and lead to materials that have a number of uses in catalysis, separation, adsorption, [5,14] as well as having the additional possibility to be converted into metal-containing porous carbonaceous materials analogous to the aforementioned Starbon^®^ materials.

In this work, we describe the preparation and characterization of a novel family of metal-containing porous starches from a range of metals including Fe, Co and Cu, denoted as FeST, CoST and CuST respectively.

## 2. Results and Discussion

**Figure 2:**
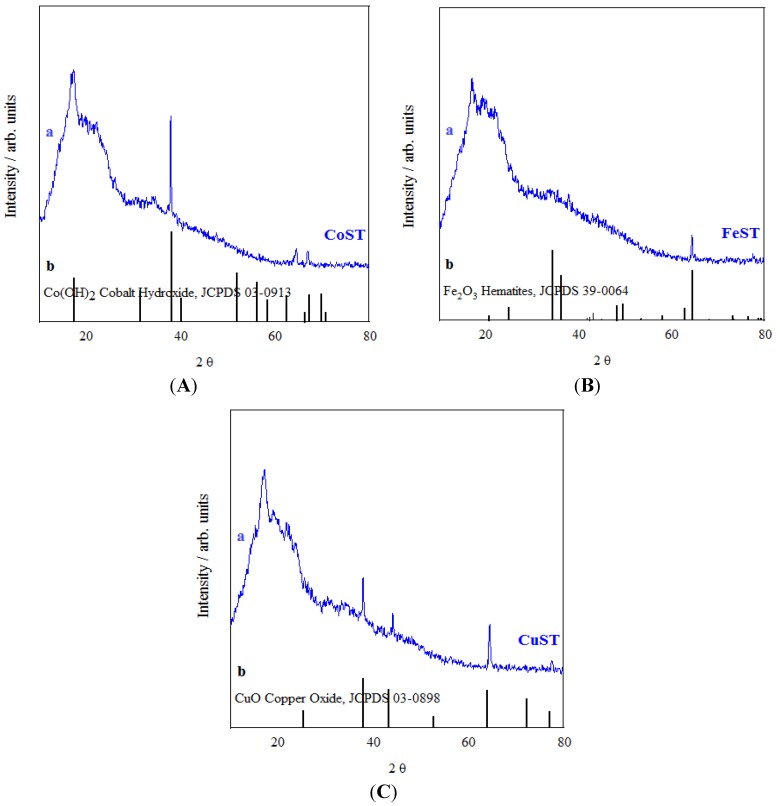
XRD diffraction patterns of (**A**) CoST; (**B**) FeST and (**C**) CuST. The bottom solid lines correspond to the metallic phases with respective JCPDS cards matching the diffraction lines found in the different materials.

The structures of porous polysaccharides synthesized in this work have been determined by means of various techniques including XRD, N_2_ physisorption and DRIFTs. XRD patterns depicted in Figure 2 show that different phases are obtained for the materials depending on the type of metal employed in the synthesis.

A characteristic broad band in the 10° to 40° range, typical of amorphous materials, was observed in all cases (Figure 2). Interestingly, metal oxide phases were exclusively present in the case of CuST and FeST derivatives, corresponding to CuO and Fe_2_O_3_ allotropic phases, respectively. Comparatively, a Co(OH)_2_ phase was obtained in CoST (Figure 2A). While no clear explanation could be found for the observed differences, the stability of both CuO and Fe_2_O_3_ phases (even at low temperatures) as compared to their respective hydroxides might be the reason for the formation of such phases, instead of the more plausible generation of hydroxide species. Low angle XRD measurements (results not shown) showed no significant information apart from the expected amorphous nature for the porous biopolymer materials, with a broad low intensity band in the 2° to 6° 2θ range.

**Figure 3:**
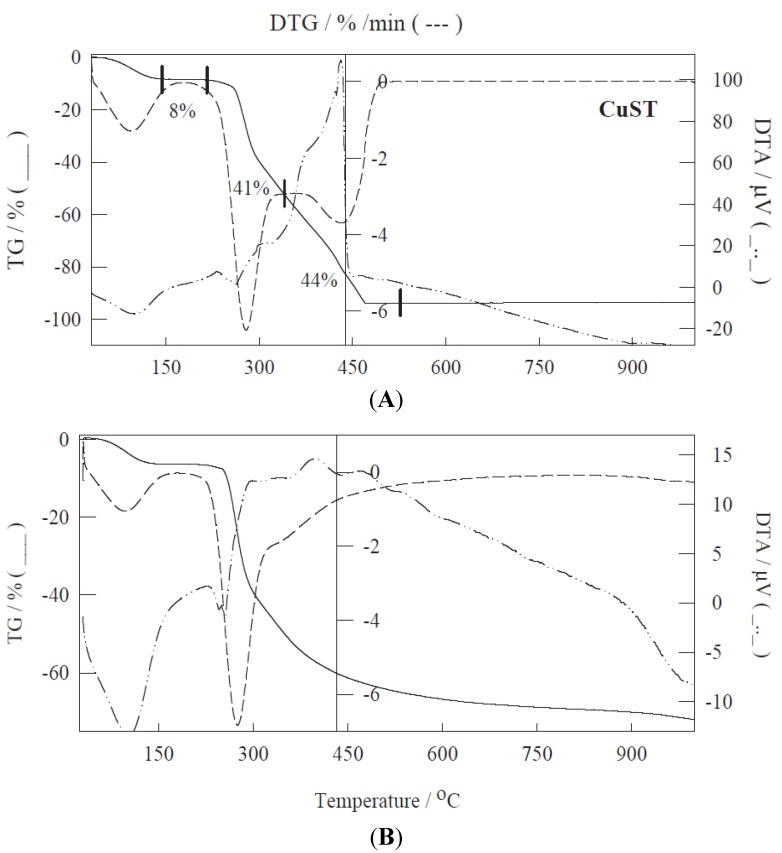
TG, DTG and DTA curves of Cu-ST under oxidizing (Air, **A**) and inert atmosphere (Ar, **B**).

TG-DTA experiments of the metal-containing starches showed a similar profile both under inert (Ar) and oxidizing atmospheres (Figure 3). These experiments are envisaged to be critical in order to determine their future suitability towards generating metal-containing Starbon^®^ materials. Four clearly distinguishable mass losses could be observed in the 30 to 900 °C range (Figure 3 CuST).

The first step at temperatures below 150 °C (*ca.* 7%–10% mass loss) can be correlated to desorption and elimination of water physisorbed and/or occluded within the pores of the materials [18]. This mass loss is accompanied by an endothermic peak in the DTA curve (Figure 3, see discontinuous lines). The second mass loss in the range of 150°–250 °C is key to the generation of Starbon^®^ materials via decomposition of the polysaccharide [6]. This step does not generally entail a significant mass loss in the materials (generally less than 2%–5%) but leads to important structural restructuration of the respective polysaccharides [1,6]. A careful temperature control in this range generally maintains their mesoporosity (see Table 1, Co-STB examples, where samples were prepared under temperature controlled and uncontrolled calcination program for comparison reason) that otherwise would collapse rendering them non-porous (Table 1). The third and fourth steps contribute to the majority of the mass loss in the material (>50% between 250 and 320 °C and *ca.* 35% between 320 and 490 °C, respectively) where the complete decomposition of the polysaccharide takes place as well as the formation of the final carbonaceous material. Of particular significance is that the TG/DTA curves are generally independent of the type of metal utilized in the synthesis but clearly dependent on the type of parent polysaccharide (e.g., starch, alginic acid, pectin, *etc.*). Different polysaccharides have different profiles and mass losses [1].

**Table 1:** Textural properties of the synthesized materials.

Materials	S_BET_^a^ (m^2^/g)	D_BJH_^b^ (nm)	V_BJH_^c^ (mL/g)
CoST	87	14.3	0.32
CuST	76	13.7	0.31
FeST	66	11.2	0.30
Co-STB-uncontrolled calcination	<5	–	–
Co-STB-controlled calcination	112	13.6	0.35

^a^ BET surface area; ^b^ mean pore size diameter and ^c^ pore volume as worked out from the Barret Joyner Halenda equation [19].

**Figure 4:**
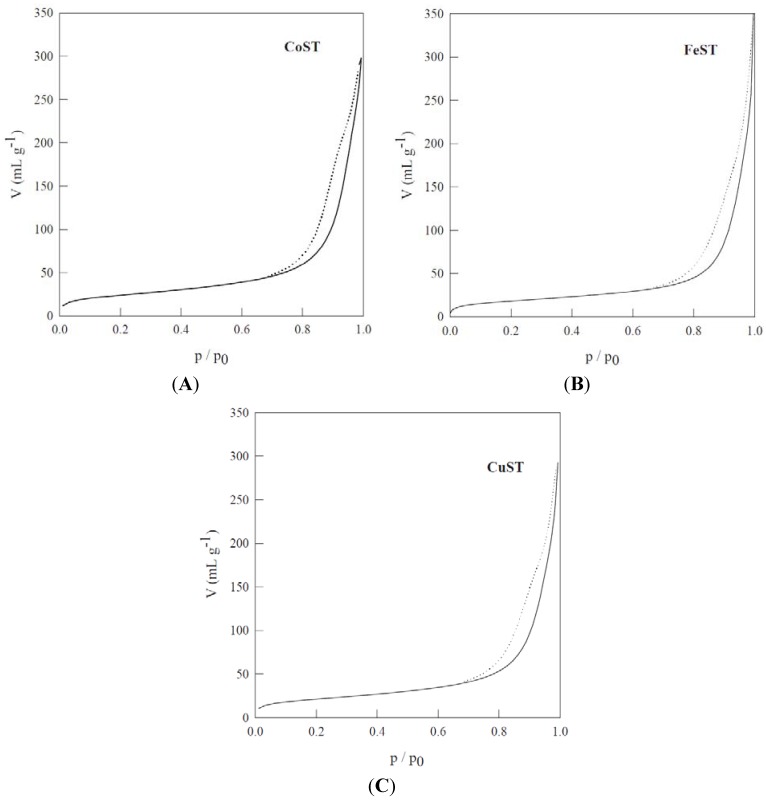
N_2_ physisorption experiments corresponding to materials: (**A**) CoST; (**B**) FeST and (**C**) CuST.

Textural properties of the materials synthesized in this work have been summarised in Table 1. Synthesized materials are predominantly mesoporous, with a broad pore size distribution in the 10–15 nm range, very different from typical well defined mesopores in ordered and well developed mesoporous materials (e.g., SBA-15). However, these metal-containing mesoporous biopolymers constitute the first report of template-free one-pot synthesised mesoporous materials derived from native non-porous starch. Some interparticular macroporosity was found to have developed in the materials at p/p_0_ > 0.98 as observable in the N_2_ physisorption isotherms (Figure 4).

Observed isotherms were of type IV with a hysteresis loop of type B, clearly dissimilar to those of conventionally ordered mesoporous materials [19], with a sharp increase in p/p_0_ from 0.85 to 0.90. Materials exhibited in general good surface areas (>60 m^2^ g^−1^) and pore volumes in the range of 0.3 mL g^−1^, particularly taking into account their template-free preparation methodology.

**Figure 5:**
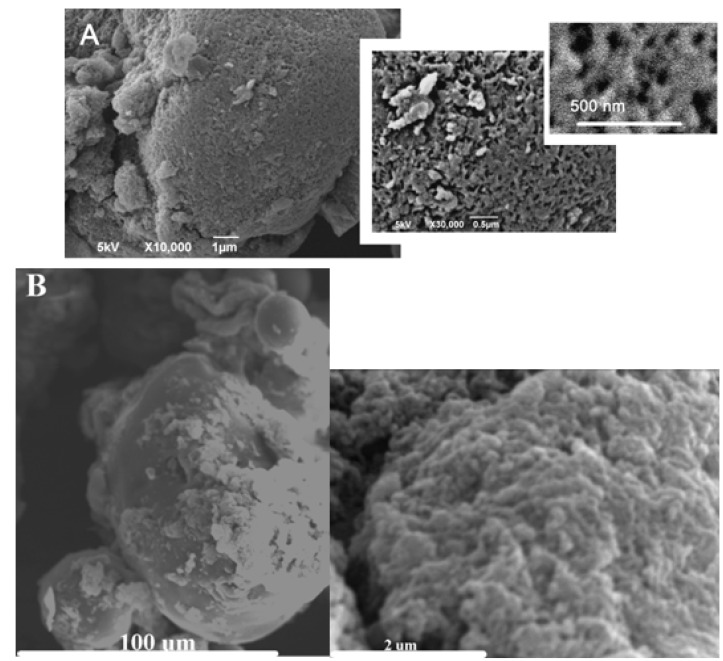
SEM micrographs of: (**A**) parent native starch and (**B**) Fe-STB.

SEM micrographs confirmed that the metal-containing polysaccharides preserved the original morphology of the parent polysaccharide, having spherical-like morphologies with particle sizes in the 1–5 µm range (Figure 5). This morphology was also preserved in the carbonized materials (especially those carbonized under inert atmosphere), in good agreement with previous reported results for Starbon^®^ related materials [1].

Table 2 summarizes metal content data for the metal-containing starch materials. These clearly show the metal incorporation was rather low (<0.2%) except for Fe which showed levels as high as 4.8% detected by ICP–MS. EDX analysis of the same material seemed to indicate that part of this Fe (almost 2%) may be within the pores of the porous polysaccharide and thus not accessible to surface analysis (as also confirmed by XPS analysis, results not shown). The low metal incorporation could be partly due to the washing and solvent exchanging steps conducted in the synthetic methodology that removed all physisorbed metals in the final materials.

**Table 2:** Actual metal content in mesoporous starches as compared to the theoretical content obtained by ICP–MS and SEM–EDX.

Materials	Theoretical metal content (%)	ICP–MS metal content (%)	SEM–EDX metal content (%)
Fe-ST	5	4.8	2.9
Co-ST	1.9	0.18	–
Cu-ST	3.9	0.2	–

**Figure 6:**
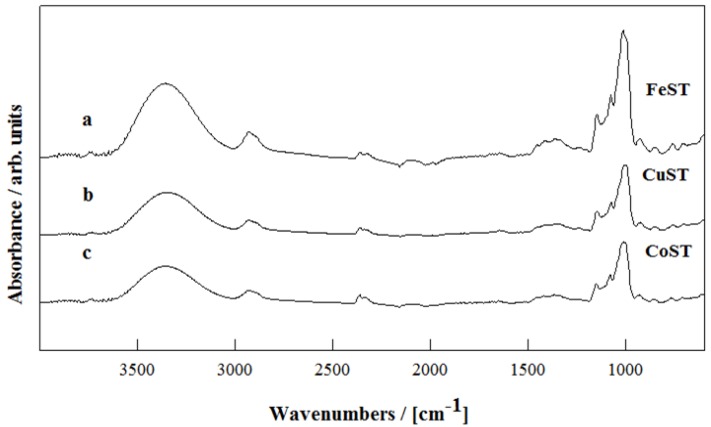
DRIFTs spectra of: (**a**) FeST; (**b**) CuST and (**c**) CoST.

**Figure 7:**
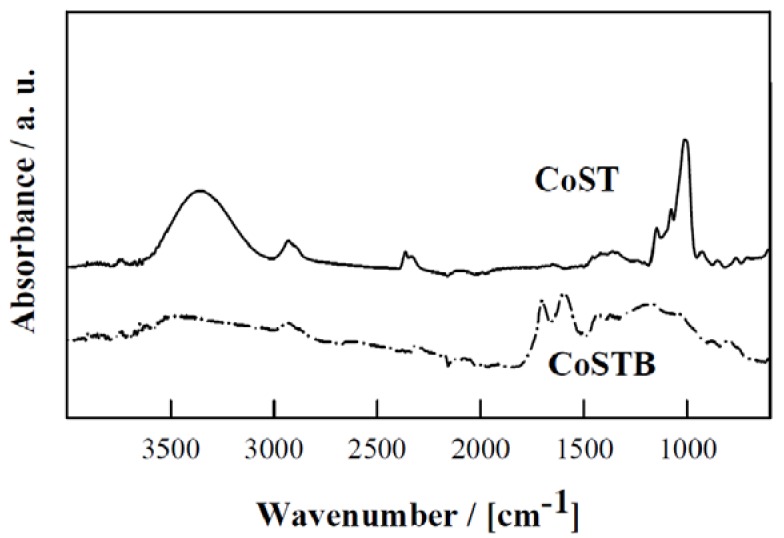
DRIFTs comparison between CuST and Cu-STB. Significant differences in functional groups were observed for both materials.

DRIFTs of all metal-containing porous starches are depicted in Figure 6, in comparison with a carbonized example shown in Figure 7. Similar spectra were obtained for all porous starch materials regardless of the metal present. Significant changes in structure upon carbonization were evidenced in the Metal-STB materials (Figure 7). Porous starches exhibited the characteristic broad bands in 3500–3200 cm^−1^ range (O–H stretching broadened via hydrogen bonding) as well as an intense band at 1050–1150 cm^−1^ (C–O stretching in ether-bonds of starch) that could be attributed to the presence of hydroxyl groups of the polysaccharide.

Upon carbonization, these bands are significantly reduced giving rise to sharp bands in the 1500–1800 cm^−1^ region due to C=C (1600–1680 cm^−1^) and C=O (1700–1750 cm^−1^) stretching bands, in good agreement with previous findings [1]. The presence of metal-oxygen bands could not be ascertained in these materials.

## 3. Experimental Section

### 3.1. Materials Synthesis

Materials synthesis involved three key steps:

1. Biopolymer expansion (key process stage), via aqueous polysaccharide gel preparation assisted by a simple and efficient microwave irradiation methodology;
2. Incorporation of the metal via addition of the metal precursor to the aqueous gel;
3. Production of porous polysaccarides, via solvent exchange/drying.

**Figure 8:**
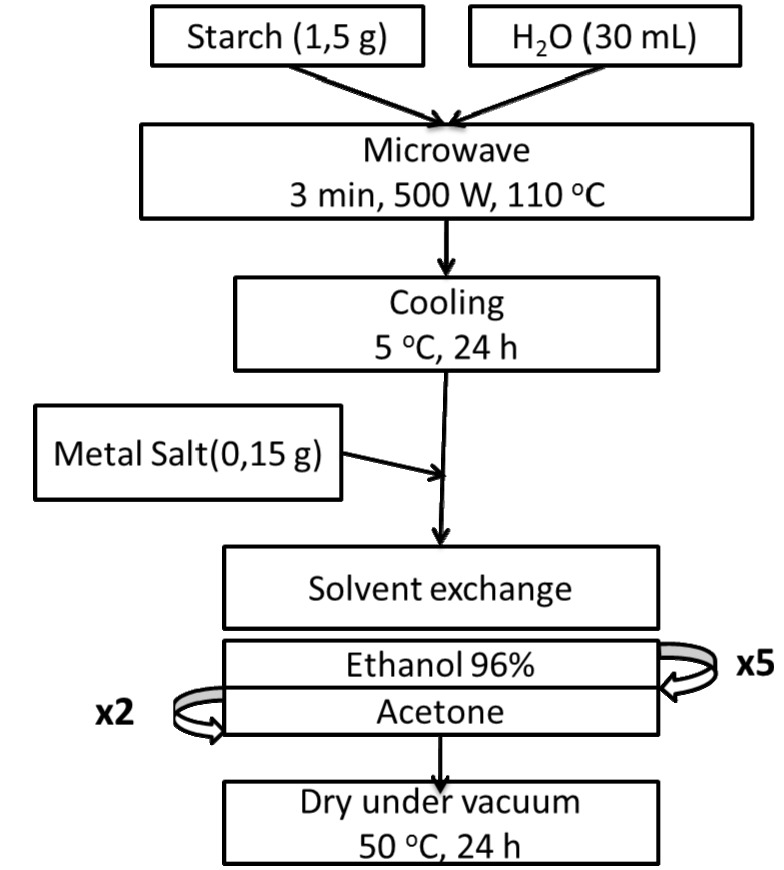
Preparation of metal-containing porous polysaccharides.

**Figure 9:**
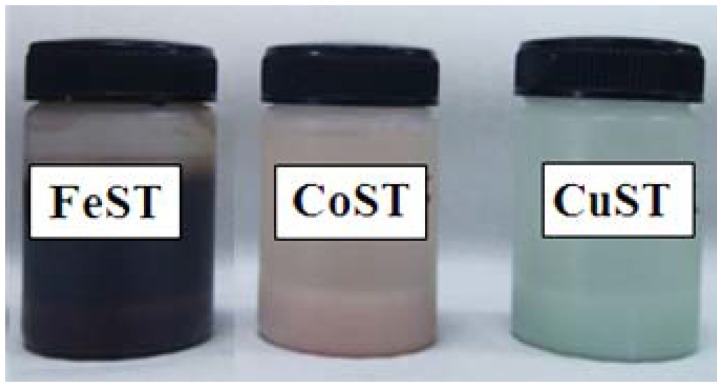
Pictorial representation of final materials, from left to right: Fe-starch (FeST), Co-starch (CoST) and Cu-starch (CuST).

A schematic representation of the process under optimized conditions has been depicted in Figure 8. Briefly, an aqueous solution of the parent native starch was microwaved for 3 min at 110 °C (500 W) in a Milestone Ethos-1 microwave multimode reactor. Reaction temperature was carefully monitored with a fiber optic probe. Upon heating, the gelatinization step yields a viscous solution that is subsequently cooled and held at 5 °C for 24 h to yield a porous gel network. Different metal salts including [Fe(C_5_H_8_O_2_)_3_], [Co(CH_3_COO)_2_·4H_2_O] and [C_2_H_2_CuO_4_·*x*H_2_O], were then added to the gel in different quantities but generally to the ratio 0.1 g metal precursor per 20 mL gel solution, and then gently stirred for a few minutes until a homogeneous phase was obtained. The gel was then solvent exchanged in a similar way to previous reports by the group [1,6] (5 times in ethanol and twice in acetone). Upon filtration, the gel was dried at 50 °C under vacuum for 24 h to yield the final metal-containing porous polysaccharides (Figure 9).

### 3.2. Materials Characterisation

X-Ray diffraction patterns (XRD) were recorded on a Siemens D-5000 (40 kV, 30 mA) diffractometer with Cu K_α_ radiation (λ = 1.54 Å). Diffractograms were collected at 0.5 min^−1^ in the 10° < 2θ < 80° range, with the aim to ascertain the different metal species present in the materials.

Nitrogen adsorption measurements were carried out at 77 K using an ASAP 2000 volumetric adsorption analyzer from Micromeritics. The samples were out-gassed for 2 h at 100 °C under vacuum (*p* < 10^−2^ Pa) and subsequently analyzed. The linear part of the BET equation (relative pressure between 0.05 and 0.30) was used for the determination of the specific surface area. Mean pore size diamete (D_BJH_) and pore volumes (V_BJH_) were obtained from porosimetry data.

Elemental composition of the calcined samples was obtained using a JEOL JSM-6300 Scanning Microscope equipped with energy-dispersive X-ray microanalysis (EDX) Inca Energy 250, detector SiLi (ATW2) at 20 kV. Detection interval: from boron to uranium, resolution 137 eV to 5.9 KeV.

Thermal analysis was performed by simultaneous thermal gravimetric and differential thermal analysis (TG-DTA) measurement using a Setsys 12 Setaram thermobalance and α-Al_2_O_3_ as the reference material and a Pt/Pt-Rh (10%) thermopar for temperature control. Samples were heated in air or argon (50 mL/min) in the 30–900 °C temperature range at a heating rate of 10 °C min^−1^.

Diffuse Reflectance Infrared Fourier-Transform (DRIFT) experiments were conducted in a Perkin Elmer Spectrum 100 Infrared Spectrometer equipped with an Attenuated Total Reflectance (ATR) module. Attenuated total reflectance infrared (FTIR-ATR) spectra of the dried polysaccharide standards were recorded using the Perkin Elmer^®^ Spectrum™ 400 FT-IR/NIR spectrometer (Perkin Elmer Inc., Tres Cantos, Madrid) in mid-IR mode, equipped with a Universal ATR (attenuated total reflectance) sampling device containing diamond/ZnSe crystal. Besides, for powdered samples an extra accessory plate with a conic awl was used which required only a few milligrams without any previous sample preparation. Spectra were acquired and then processed with the Spectrum software version 6.3.2. The spectra were scanned at room temperature in absorbance mode over the wave number range of 4000–650 cm^−1^, with a scan speed of 0.20 cm/s, and 30 accumulations at a resolution of 4 cm^−1^. Materials were dried at 100 °C for 3 h prior to measurement.

ICP-MS analysis was conducted at the Servicios Centrales de Apoyo a la Investigación (SCAI), Universidad de Cordoba using an ICP-MS ELAN-DRC-e (Perkin Elmer). Materials were dissolved in a HNO_3_:HCl:HF mixture (5 mL, 2:2:1 ratio).

## 4. Conclusions

A new family of metal-containing mesoporous starches have been developed using a simple microwave-assisted methodology comprising gelation of the parent polysaccharide followed by incorporation of the metals and subsequent solvent exchange and drying. Mesoporous materials with high surface areas (>60 m^2^·g^−1^) could be obtained by means of the proposed protocol that have the potential to be further converted into metal-containing Starbon^®^ materials with different structures and compositions depending on the temperature of carbonization. Potentially interesting metal phases were observed, including metal oxides and hydroxides in the materials that are envisaged to have promising catalytic applications (e.g., heterogeneous catalysis and environmental remediation).
